# Supplementary material for: Synthesis of a Cyclooctapeptide, Cyclopurpuracin, and Evaluation of Its Antimicrobial Activity
Source: Molecules. 2023 Jun 15;28(12):4779. doi: 10.3390/molecules28124779 (PMC10301653; doi:10.3390/molecules28124779)
Supplement: Supplementary file 1 [file molecules-28-04779-s001.zip › molecules-2438747-supplementary.pdf]

SUPPLEMENTARY MATERIALS

Synthesis of a cyclooctapeptide, cyclopurpuracin, and evaluation of its antimicrobial activity

Supplementary Figures

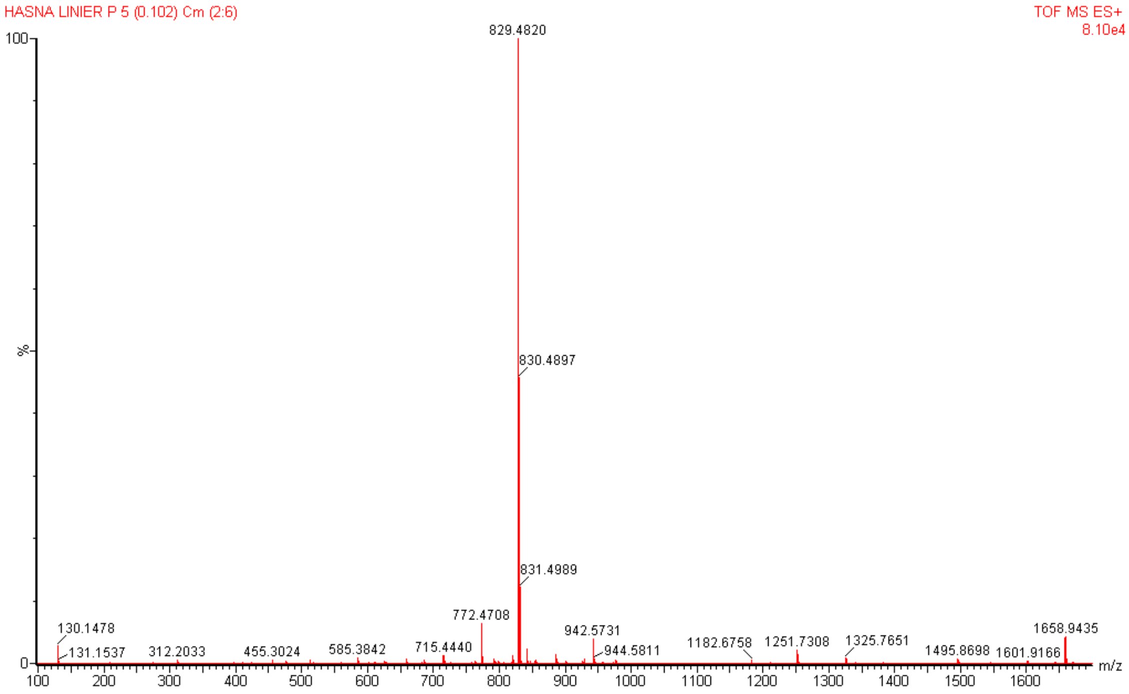

Figure S1. MS spectra of linear precursor A **3** (precursor of cyclopurpuracin).

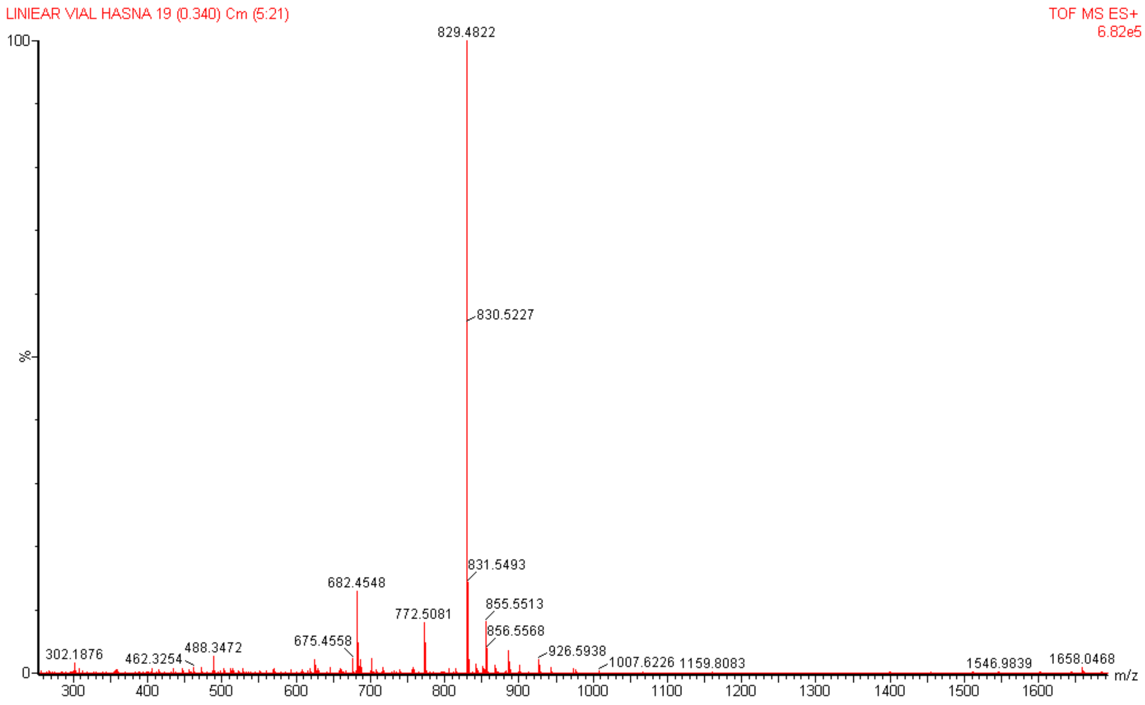

Figure S2. MS spectra of linear precursor B **3** (precursor of cyclopurpuracin).

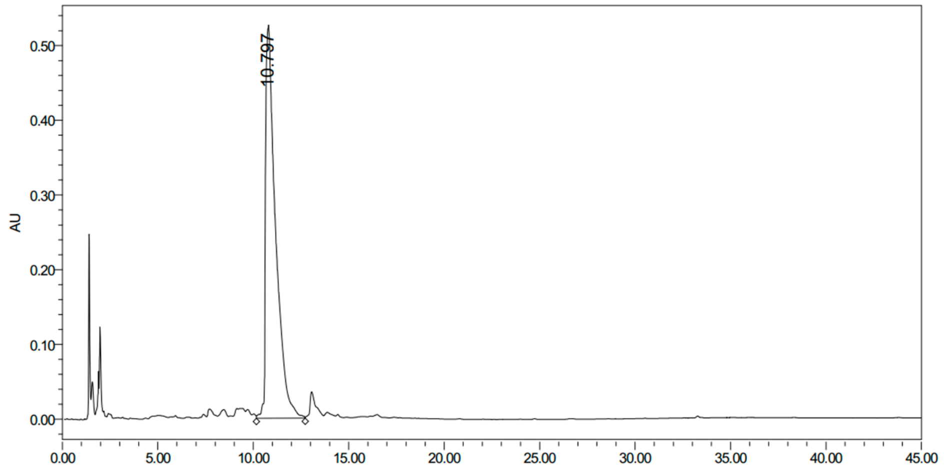

Figure **S3**. Analytical RP-HPLC chromatogram of linear precursor A **3** (precursor of cyclopurpuracin) in acetonitrile: H_2O (linear gradient, 20:80-80:20), flow rate (1 mL/min), λ 210 nm.

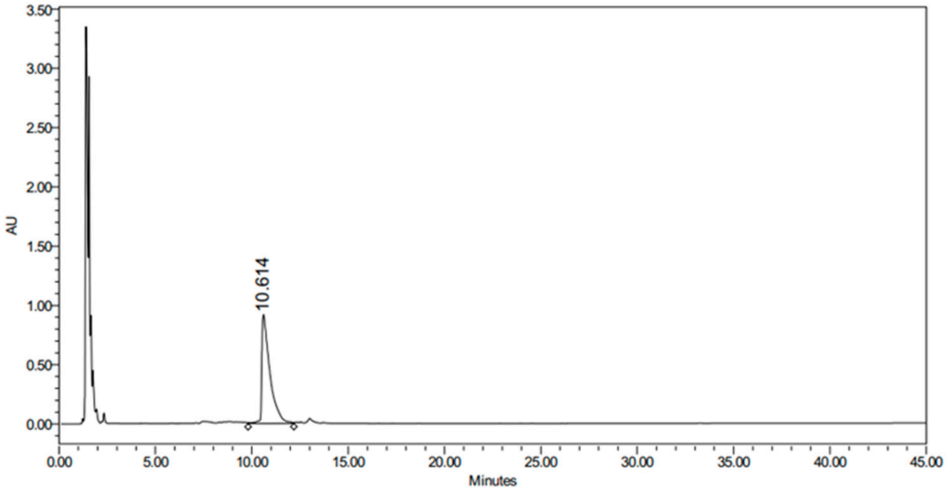

Figure **S4**. Analytical RP-HPLC chromatogram of linear precursor B **3** (precursor of cyclopurpuracin) in acetonitrile: H_2O (linear gradient, 20:80-80:20), flow rate (1 mL/min), λ 210 nm.

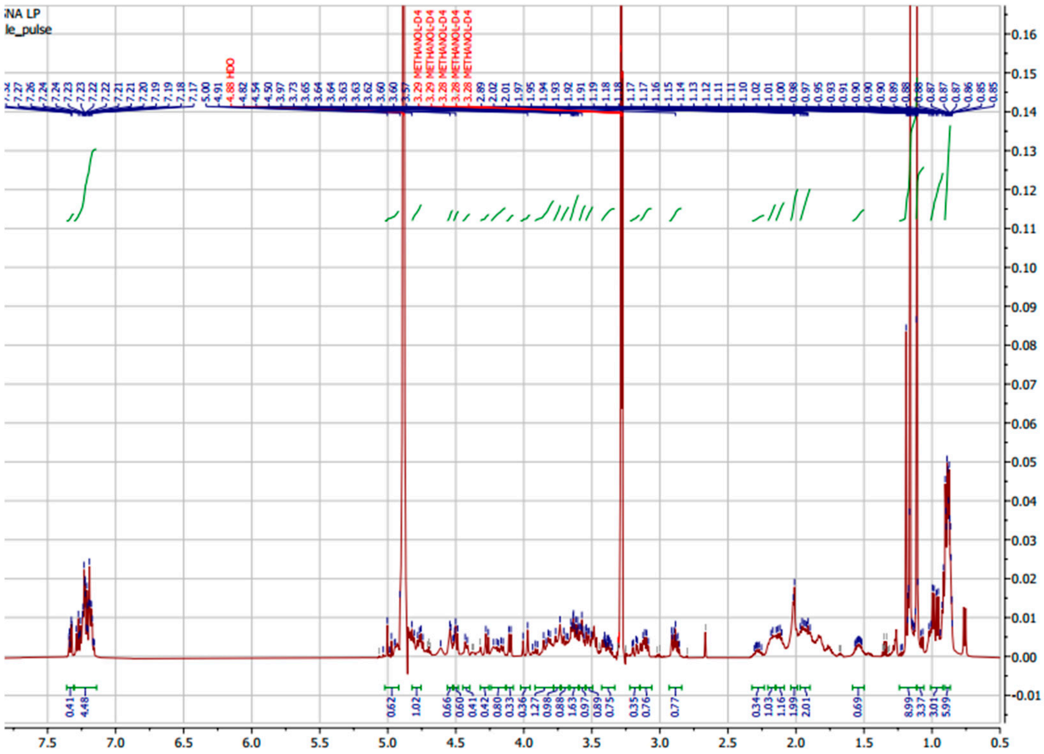

Figure **S5**. ^{1}H NMR spectra of precursor linear A **3** (precursor linear of cyclopurpuracin)

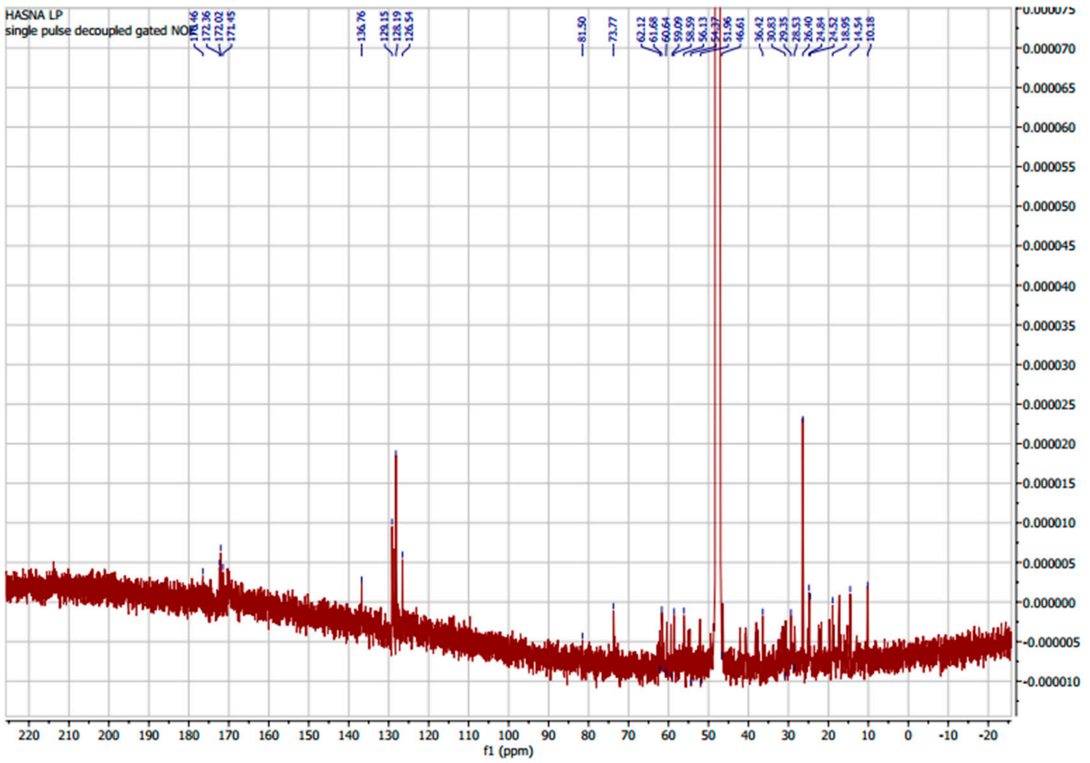

Figure **S6**. ^{13}C NMR spectra of precursor linear A **3** (precursor linear of cyclopurpuracin)

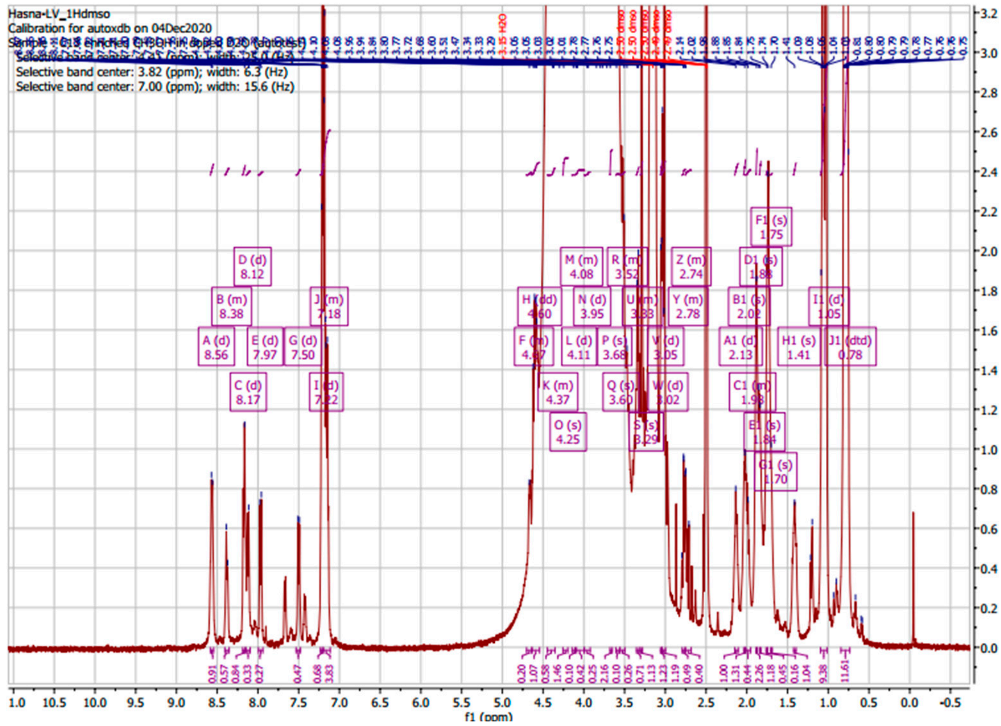

Figure **S7**. ^{1}H NMR spectra of precursor linear B **3** (precursor linear of cyclopurpuracin)

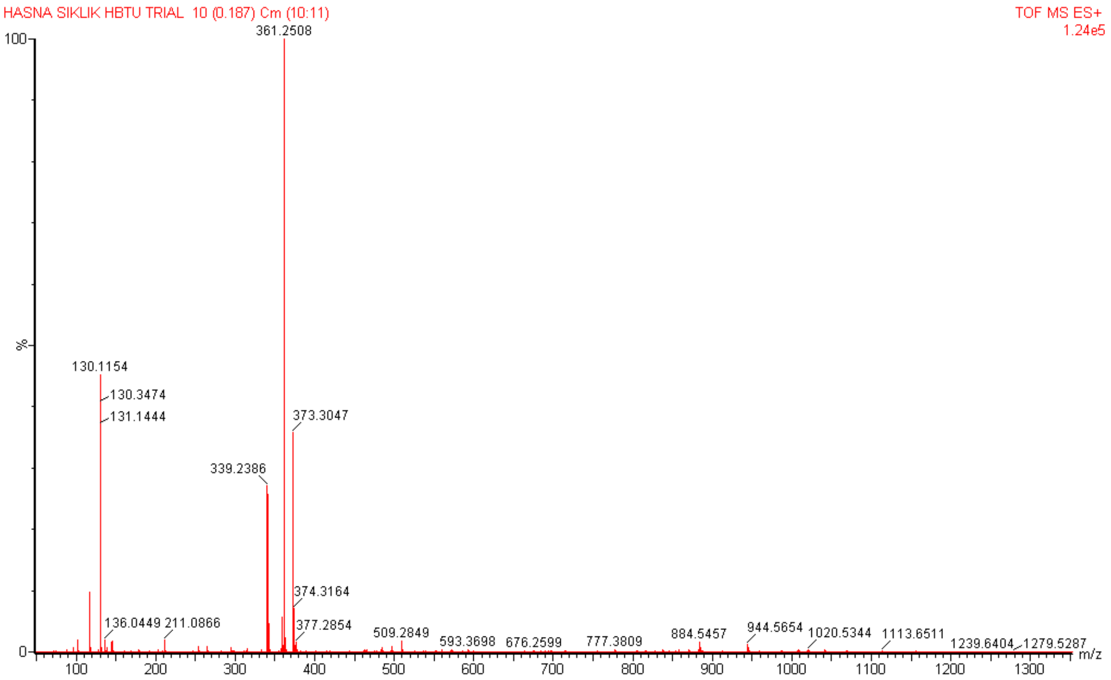

Figure S8. MS spectra of cyclopurpuracin **1** cyclized using HBTU.

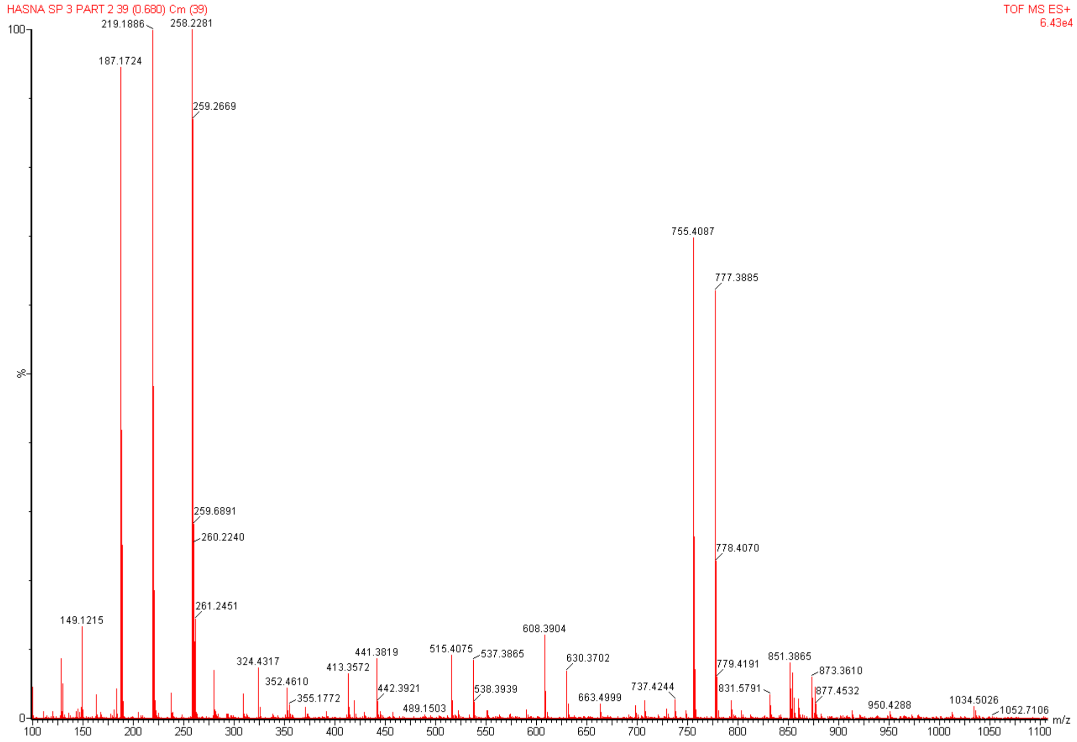

Figure S9. MS spectra of crude cyclopurpuracin **1** from precursor linear A using PyBOP/NaCl.

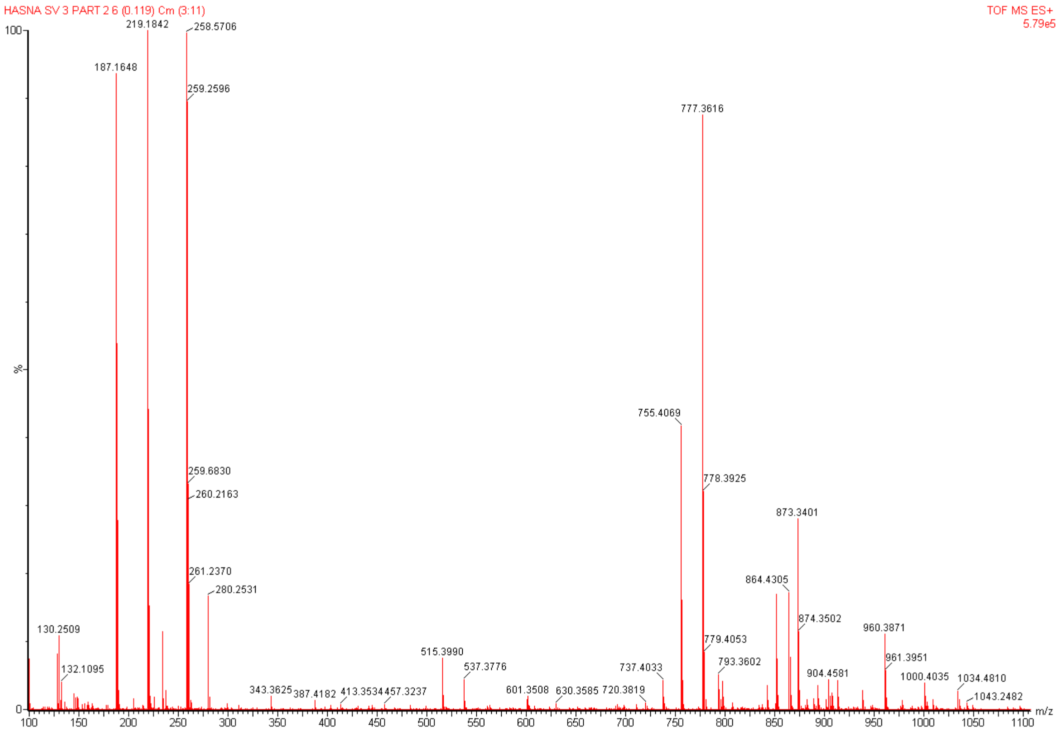

Figure S10. MS spectra of crude cyclopurpuracin **1** from precursor linear B using PyBOP/NaCl.

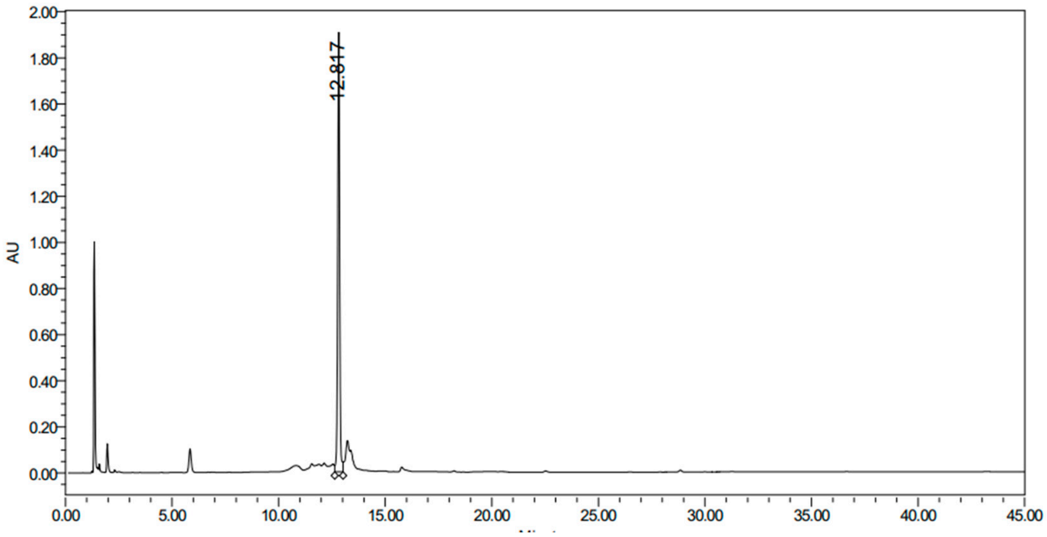

Figure **S11**. Analytical RP-HPLC chromatogram of cyclopurpuracin **1** from precursor linear A using PyBOP/NaCl in acetonitrile: H_2O (linear gradient, 20:80-80:20), flow rate (1 mL/min), λ 210 nm.

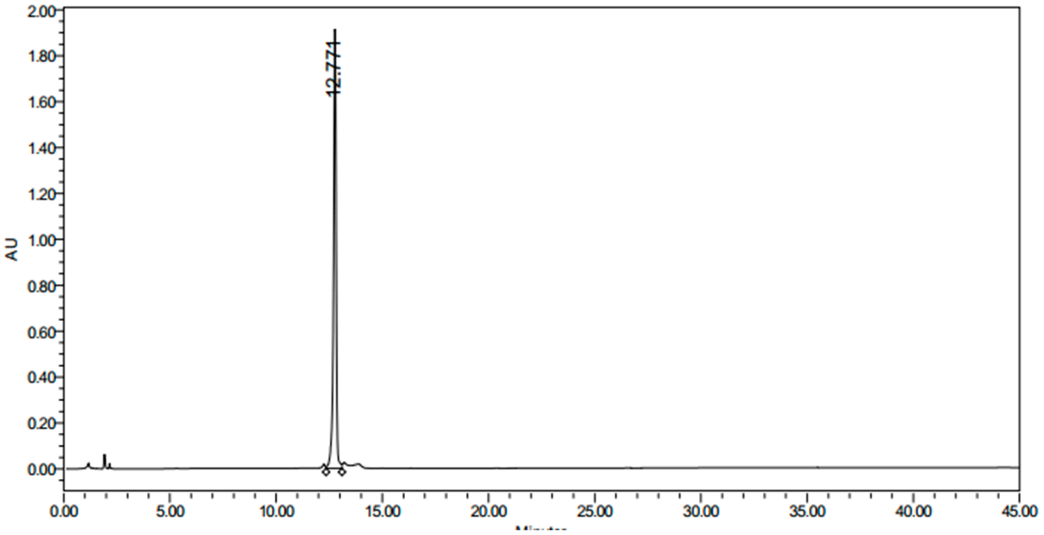

Figure **S12**. Analytical RP-HPLC chromatogram of cyclopurpuracin **1** from precursor linear A using PyBOP/NaCl in acetonitrile: H_2O (linear gradient, 20:80-80:20), flow rate (1 mL/min), λ 210 nm.

.

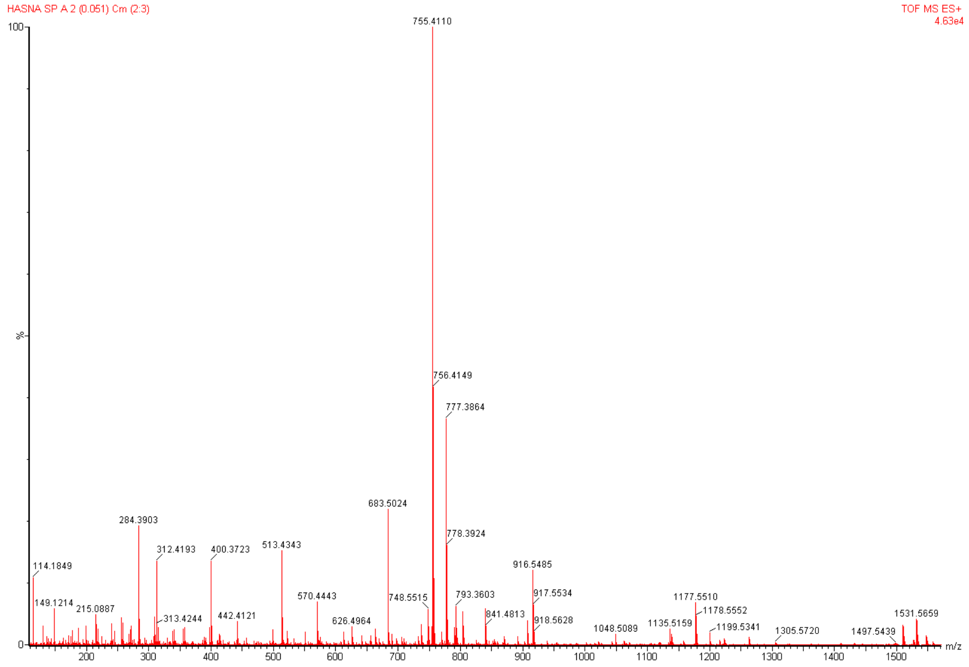

Figure S13. MS spectra of cyclopurpuracin **1** from precursor linear A using PyBOP/NaCl.

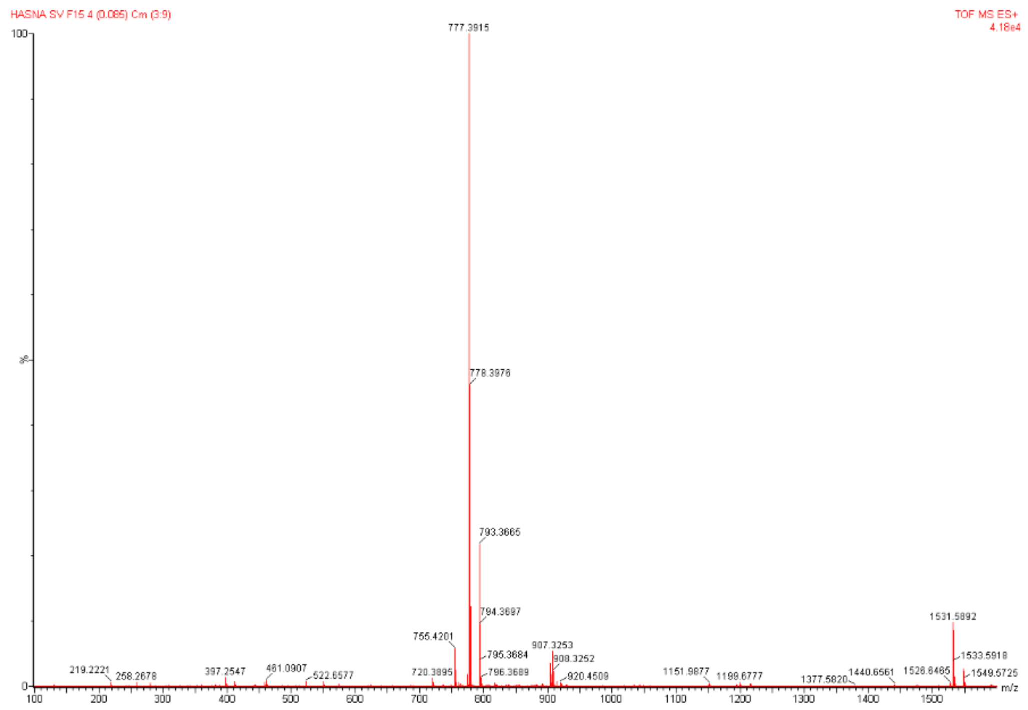

Figure S14. MS spectra of cyclopurpuracin **1** from precursor linear B using PyBOP/NaCl.

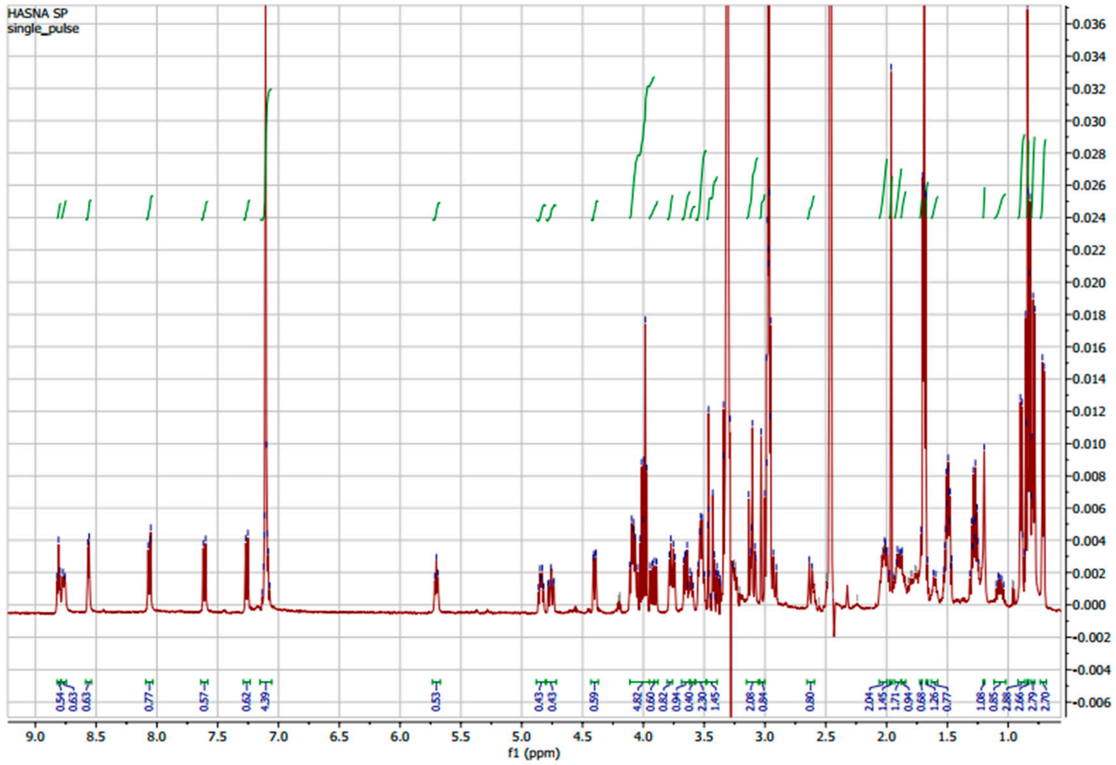

Figure **S15.** ^{1}H NMR spectra of cyclopurpuracin **1** from precursor linear A using PyBOP/NaCl.

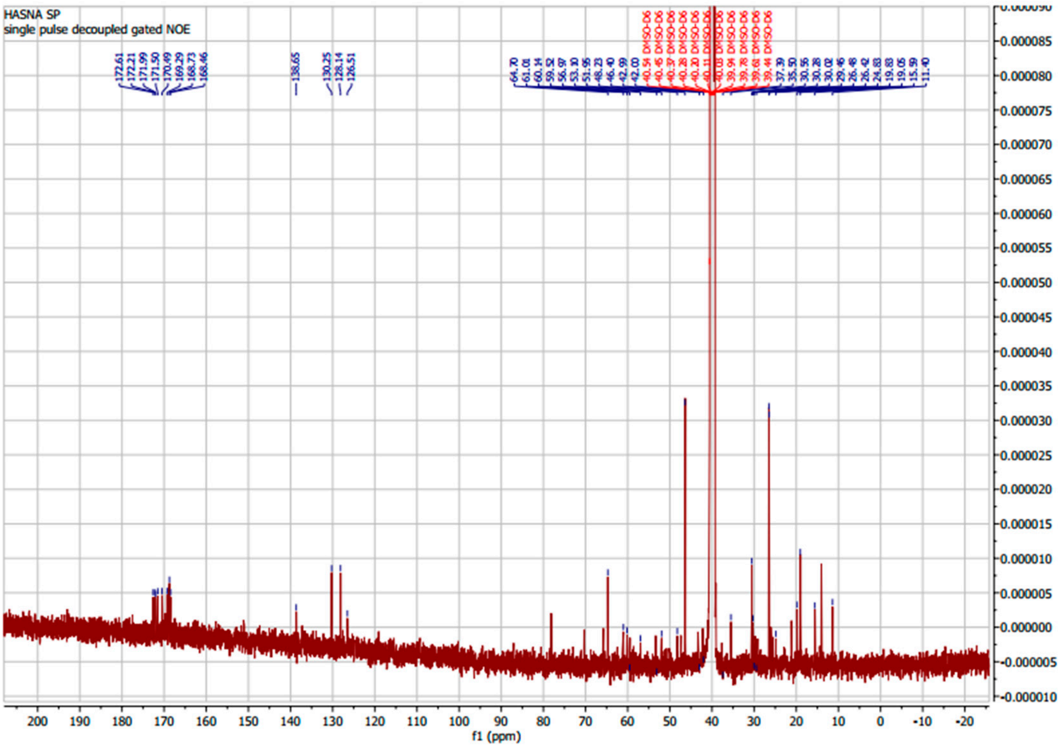

Figure **S16.** ^{13}C NMR spectra of cyclopurpuracin **1** from precursor linear A using PyBOP/NaCl.

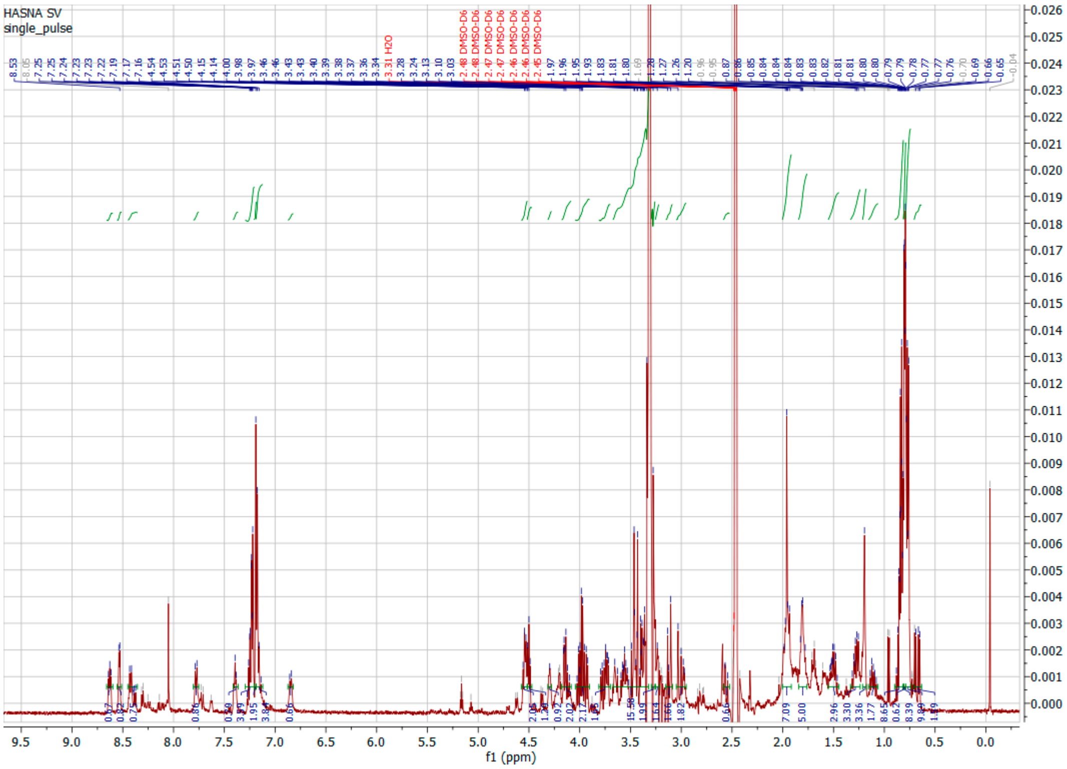

Figure **S17.** ^{1}H NMR spectra of cyclopurpuracin **1** from precursor linear B using PyBOP/NaCl.

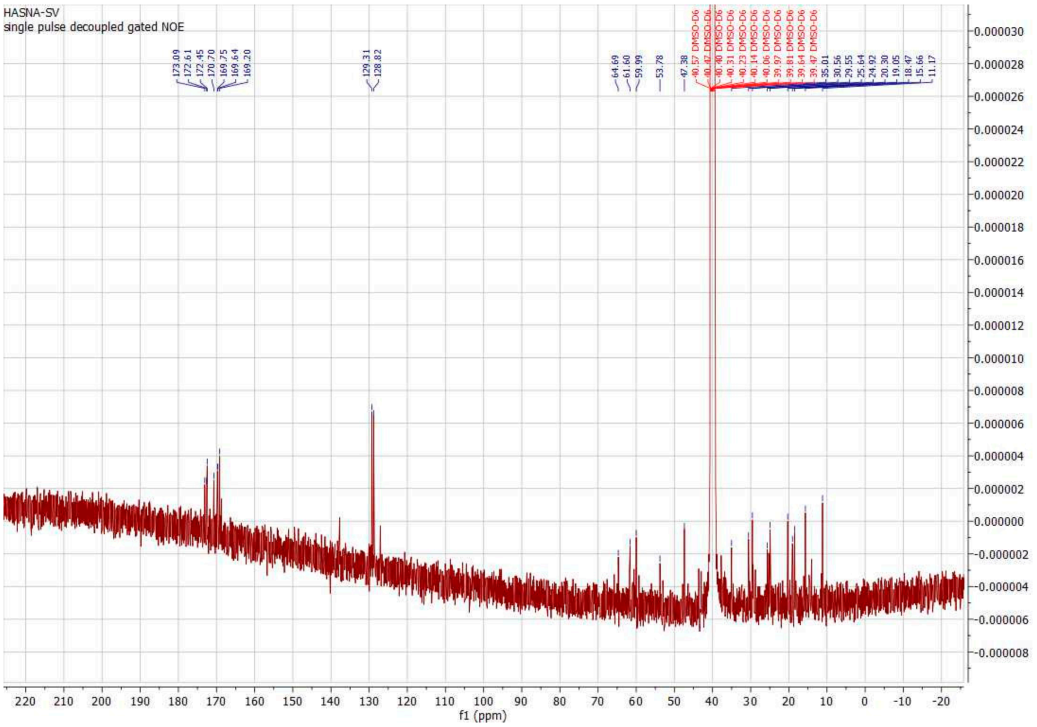

Figure **S18.** ^{13}C NMR spectra of cyclopurpuracin **1** from precursor linear B using PyBOP/NaCl.

Supplementary table

Table S1. Chemical shift differences between cyclopurpuracin from precursor linear A and isolated cyclopurpuracin

Assignment	Cyclopurpuracin from precursor linear A		Isolated cyclopurpuracin [9]		Δδ (ppm)	
	^{1}H δ in ppm (ΣH, m)	^{13}C (ppm)	^{1}H δ in ppm (ΣH, m)	^{13}C (ppm)	^{1}H	^{13}C
**Pro¹**						
CO		172.2		172.2		-
α-CH	4.10 (1H, d)	61.0	4.15 (1H, d)	61.6	0.05	0.6
β-CH_2	1.79 (2H, m)	29.7	1.79 (2H, m)	29.5	-	0.2
γ-CH_2	2.01 (2H, m)	25.2	2.08 (2H, m)	25.2	0.07	-
δ-CH_2	4.06 (2H, m)	48.2	4.10 (2H, m)	48.1	0.04	0.1
**Gly²**						
CO		168.7		168.8		0.1
α-CH	4.02; 3.10 (1H, dd)	42.2	3.97; 3.14 (1H, dd)	42.9	0.05; 0.04	0.7
NH	8.77 (1H, dd)		8.78 (1H, dd)		0.01	
**Phe³**						
CO		171.9		171.9		0.5
α-CH	4.76 (1H, m)	53.3	4.81 (1H, ddd)	53.3	0.05	-
β-CH_2	2.62; 3.03 (2H, dd)	37.6	2.68; 2.97 (2H, dd)	37.7	0.06;0.06	0.1
C_1 (ar.)		138.6		138.6		-
$C_{2.6}$ (ar.)	7.11 (2H, m)	130.2	7.15 (2H, m)	130.1	0.04	0.1
$C_{3.5}$ (ar.)	7.13 (2H, m)	128.1	7.16 (2H,m)	128.0	0.03	0.1
C_4 (ar.)	7.09 (1H, m)	126.5	7.13 (1H, d)	126.4	0.04	0.1
NH	7.61 (1H, d)		7.65 (1H, d)		0.04	
**Ile⁴**						
CO		172.6		172.4		0.2
α-CH	3.92 (1H, m)	59.4	3.71 (1H, m)	59.4	0.21	-
β-CH_2	1.68 (1H, m)	35.4	1.66 (1H, ddt)	35.5	0.02	0.1
CH_3- β	0.82 (3H, d)	15.5	0.84 (3H, d)	15.5	0.02	-
γ-CH	1.07; 1.61 (2H, m)	26.4	1.12; 1.56 (2H, m)	25.9	0.05;0.05	0.5
δ-CH_3	0.84 (3H, t)	11.4	0.87 (3H, t)	11.3	0.03	0.1
NH	8.56 (1H, d)		8.58 (1H, d)		0.02	
**Gly⁵**						
CO		168.4		168.3		0.1
α-CH	3.98; 3.42 (2H, d)	43.3	3.80; 3.45 (2H, d)	43.3	0.18;0.03	-
NH	8.80 (1H, t)		8.83 (1H, t)		0.03	
**Ser⁶**						
CO		171.5		171.3		0.2
α-CH	4.84 (1H, dt)	51.9	4.89 (1H, dt)	51.9	0.05	-
β-CH	3.76; 3.60 (2H, m)	64.7	3.66; 3.57 (2H, m)	64.5	0.1;0.03	0.2
OH	5.70 (1H, t)		5.71 (1H, t)		0.1	
NH	7.26 (1H, dt)		7.31 (1H, dt)		0.05	
**Pro⁷**						
CO		171.5		171.3		0.2
α-CH	4.40 (1H, dd)	60.1	4.45 (1H, dd)	60.1	0.05	-
β-CH_2	1.91 (2H, m)	29.7	1.94 (2H, m)	29.1	0.03	0.6

γ-CH_2	1.87; 1.70 (2H, m)	24.8	1.90; 1.74 (2H, m)	24.8	0.03;0.04	-
δ-CH_2	4.00; 3.65 (2H, m)	47.3	3.83; 3.60 (2H, m)	47.3	0.17;0.05	-
**Val[8]**						
CO		170.4		170.4		-
α-CH	4.08 (1H, m)	56.9	4.12 (1H, m)	56.9	0.04	-
β-CH	1.96 (1H, m)	30.2	1.97 (1H, m)	29.1	0.01	1.1
γ-CH_3	0.88 (3H, d)	19.0	0.94 (3H, d)	19.0	0.06	-
γ-CH_3	0.71 (3H, d)	19.8	0.76 (3H, d)	19.8	0.05	-
NH	8.06 (1H, d)		8.09 (1H, d)		0.03	

Table S2. Chemical shift differences between cyclopurpuracin from precursor linear B and isolated cyclopurpuracin

Assignment	Cyclopurpuracin from precursor linear B		Isolated cyclopurpuracin [9]		Δδ (ppm)	
	^{1}H δ in ppm (ΣH, m)	^{13}C (ppm)	^{1}H δ in ppm (ΣH, m)	^{13}C (ppm)	^{1}H	^{13}C
**Pro[1]**						
CO		172.6		172.2		0.4
α-CH	4.37 (1H, d)	60.9	4.15 (1H, d)	61.6	0.22	0.7
β-CH_2	1.79 (2H, m)	29.7	1.79 (2H, m)	29.5	-	0.2
γ-CH_2	1.98 (2H, m)	25.2	2.08 (2H, m)	25.2	0.1	-
δ-CH_2	4.16 (2H, m)	48.1	4.10 (2H, m)	48.1	0.06	-
**Gly[2]**						
CO		169.2		168.8		0.4
α-CH	4.11;3.12 (1H, dd)	42.1	3.97; 3.14 (1H, dd)	42.9	0.14; 0.02	0.8
NH	8.53(1H, d)		8.78 (1H, dd)		0.25	
**Phe[3]**						
CO		172.4		171.9		0.5
α-CH	4.55 (1H, m)	53.7	4.81 (1H, ddd)	53.3	0.26	0.4
β-CH_2	2.59; 3.00 (2H, m)	37.8	2.68; 2.97 (2H, dd)	37.7	0.09; 0.03	0.1
C_1 (ar.)		137.2		138.6		1.4
$C_{2.6}$ (ar.)	7.20 (2H, m)	128.8	7.15 (2H, m)	130.1	0.05	1.3
$C_{3.5}$ (ar.)	7.20 (2H, m)	129.3	7.16 (2H,m)	128.0	0.04	1.3
C_4 (ar.)	7.24 (2H, m)	127.0	7.13 (1H, d)	126.4	0.11	0.6
NH	7.75 (1H, dd)		7.65 (1H, d)		0.1	
**Ile[4]**						
CO		173.0		172.4		0.6
α-CH	3.93 (1H, m)	59.5	3.71 (1H, m)	59.4	0.22	0.1
β-CH_2	1.50 (1H, m)	36.9	1.66 (1H, ddt)	35.5	0.16	1.4
CH_{3}- β	0.79 (3H, d)	15.6	0.84 (3H, d)	15.5	0.05	0.1
γ-CH	1.11; 1.28 (2H, m)	25.6	1.12; 1.56 (2H, m)	25.9	0.01;0.28	0.3
δ-CH_3	0.82 (3H, t)	11.1	0.87 (3H, t)	11.3	0.05	0.2
NH	8.40 (1H, dd)		8.58 (1H, d)		0.18	
**Gly[5]**						
CO		168.7		168.3		0.4
α-CH	4.00; 3.45 (2H, d)	43.6	3.80; 3.45 (2H, d)	43.3	0.2;-	0.3

NH	8.63 (1H, t)		8.83 (1H, t)		0.2	
**Ser[6]**						
CO		169.6		171.3		1.7
α-CH	4.62 (1H, d)	53.7	4.89 (1H, dt)	51.9	0.27	1.8
β-CH	3.79; 3.57 (2H, m)	64.6	3.66; 3.57 (2H, m)	64.5	0.13;-	0.1
OH	5.16 (1H, t)		5.71 (1H, t)		0.55	
NH	7.39(1H, d)		7.31 (1H, dt)		0.08	
**Pro[7]**						
CO		170.7		171.3		0.6
α-CH	4.50 (1H, d)	60.3	4.45 (1H, dd)	60.1	0.05	0.2
β-CH_2	1.93 (2H, m)	29.5	1.94 (2H, m)	29.1	0.01	0.4
γ-CH_2	1.82; 1.69 (2H, m)	24.9	1.90; 1.74 (2H, m)	24.8	0.08;0.05	0.1
δ-CH_2	3.96; 3.75 (2H, m)	47.3	3.83; 3.60 (2H, m)	47.3	0.13;0.15	-
**Val[8]**						
CO		169.7		170.4		0.7
α-CH	4.30 (1H, m)	59.2	4.12 (1H, m)	56.9	0.08	2.3
β-CH	1.96 (1H, m)	30.5	1.97 (1H, m)	29.1	0.01	1.4
γ-CH_3	0.88 (3H, d)	19.0	0.94 (3H, d)	19.0	0.06	-
γ-CH_3	0.76 (3H, d)	20.2	0.76 (3H, d)	19.8	-	0.4
NH	8.07 (1H, dd)		8.09 (1H, d)		0.02	
